# Tebuconazole and penconazole – Determination of TEB-OH, TEB-COOH, PEN-OH, and PEN-COOH in urine by LC-MS/MS: Biomonitoring Method – Translation of the German version from 2026

**DOI:** 10.34865/bi10753496e11_1or

**Published:** 2026-03-30

**Authors:** Elisa Polledri, Rosa Mercadante, Silvia Fustinoni, Laura Kuhlmann, Thomas Göen, Andrea Hartwig, MAK Commission

**Affiliations:** 1 Laboratory of Environmental and Occupational Toxicology. Department of Clinical Sciences and Community Health. University of Milano and Fondazione IRCCS Ca Granda Ospedale Maggiore Policlinico Via Francesco Sforza 35 20122 Milano Italy; 2 Friedrich-Alexander-Universität Erlangen-Nürnberg. Institute and Outpatient Clinic of Occupational, Social, and Environmental Medicine Henkestraße 9–11 91054 Erlangen Germany; 3 Institute of Applied Biosciences. Department of Food Chemistry and Toxicology. Karlsruhe Institute of Technology (KIT) Adenauerring 20a, Building 50.41 76131 Karlsruhe Germany; 4 Permanent Senate Commission for the Investigation of Health Hazards of Chemical Compounds in the Work Area. Deutsche Forschungsgemeinschaft, Kennedyallee 40, 53175 Bonn, Germany. Further information: Permanent Senate Commission for the Investigation of Health Hazards of Chemical Compounds in the Work Area | DFG

**Keywords:** tebuconazole, penconazole, biomonitoring, urine, LC-MS/MS

## Abstract

The working group “Analyses in Biological Materials” of the German Senate Commission for the Investigation of Health Hazards of Chemical Compounds in the Work Area (MAK Commission) developed and verified this biomonitoring method for the measurement of the most important urinary metabolites of the triazole fungicides tebuconazole and penconazole. Specifically, this method determines (*RS*)-5-(4-chlorophenyl)-2,2-dimethyl-3-(1*H*-1,2,4-triazol-1-ylmethyl)-1,3-pentanediol (TEB-OH) and (*RS*)-5-(4-chlorophenyl)-2,2-dimethyl-3-(1*H*-1,2,4-triazol-1-ylmethyl)-3-olpentanoic acid (TEB-COOH) as well as 4-(2,4-dichlorophenyl)-5-[1,2,4]-triazol-1-ylpentanol (PEN-OH) and 4-(2,4-dichlorophenyl)-5-[1,2,4]-triazol-1-ylpentanoic acid (PEN-COOH) in urine. After adding isotope-labelled internal standards, the samples are enzymatically hydrolysed to release the analytes from the glucuronide and sulfate conjugates. After online purification, the analytes are separated by liquid chromatography and analysed using tandem mass spectrometry. Calibration is performed using calibration standards prepared in pooled urine and processed analogously to the samples to be analysed. The method provides reliable and accurate analytical results, as shown by the good precision data with standard deviations in the range of 0.1–10.7%. Good accuracy data were obtained with mean relative recoveries in the range of 100–107%. The method is selective, sensitive, and provides quantitation limits of 0.3 μg/l for TEB-OH and TEB-COOH and of 1.0 μg/l for PEN-OH and PEN-COOH.

## 1 Characteristics of the method

**Table:** 

**Matrix**	Urine		
**Analytical principle**	Liquid chromatography with tandem mass spectrometry (LC‑MS/MS)		
**Parameters and corresponding hazardous substances**			
**Hazardous substance**	**CAS No.**	**Parameter**	**CAS No.**
Tebuconazole (1‑(4‑Chlorophenyl)-4,4‑dimethyl-3‑(1*H*‑1,2,4‑triazol-1‑ylmethyl)-pentan‑3‑ol)	107534‑96‑3	(*RS*)‑5‑(4‑Chlorophenyl)-2,2‑dimethyl-3‑(1*H*‑1,2,4‑triazol-1‑ylmethyl)-1,3‑pentanediol (TEB‑OH)	212267‑64‑6
		(*RS*)-5‑(4‑Chlorophenyl)-2,2‑dimethyl-3‑(1*H*‑1,2,4‑triazol-1‑ylmethyl)-3‑olpentanoic acid (TEB‑COOH)	1631977-20-2
Penconazole ((*RS*)-1‑[2‑(2,4‑Dichlorophenyl)-pentyl]‑1*H*‑1,2,4‑triazole)	66246‑88‑6	4‑(2,4‑Dichlorophenyl)-5‑[1,2,4]-triazol-1‑ylpentanol (PEN‑OH)	1945983-65-2
		4‑(2,4‑Dichlorophenyl)-5‑[1,2,4]‑triazol-1‑ylpentanoic acid (PEN‑COOH)	1945983-66-3

### Reliability data

#### TEB‑OH

**Table:** 

Within-day precision:	Standard deviation (rel.)	*s_w_* = 0.1–1.7% or 0.1–1.4%
	Prognostic range	*u* = 0.28–4.72% or 0.28–3.89%
	at a spiked concentration of 20 μg or 150 μg TEB‑OH per litre of urine and n = 5 determinations	
Day-to-day precision:	Standard deviation (rel.)	*s_w_* = 1.0% or 0.8%
	Prognostic range	*u* = 2.45% or 1.96%
	at a spiked concentration of 20 μg or 150 μg TEB‑OH per litre of urine and n = 7 determinations	
Accuracy:	Recovery (rel.)	*r* = 100% or 100%
	at a spiked concentration of 20 μg or 150 μg TEB‑OH per litre of urine and n = 5 determinations	
Limit of detection:	0.2 μg TEB‑OH per litre of urine	
Limit of quantitation:	0.3 μg TEB‑OH per litre of urine	

#### TEB‑COOH

**Table:** 

Within-day precision:	Standard deviation (rel.)	*s_w_* = 0.4–3.1% or 0.1–0.9%
	Prognostic range	*u* = 1.11–8.61% or 0.28–2.50%
	at a spiked concentration of 8 μg or 60 μg TEB‑COOH per litre of urine and n = 5 determinations	
Day-to-day precision:	Standard deviation (rel.)	*s_w_* = 1.6% or 1.4%
	Prognostic range	*u* = 3.92% or 3.43%
	at a spiked concentration of 8 μg or 60 μg TEB‑COOH per litre of urine and n = 7 determinations	
Accuracy:	Recovery (rel.)	*r* = 100% or 101%
	at a spiked concentration of 8 μg or 60 μg TEB‑COOH per litre of urine and n = 5 determinations	
Limit of detection:	0.1 μg TEB‑COOH per litre of urine	
Limit of quantitation:	0.3 μg TEB‑COOH per litre of urine	

#### PEN‑OH

**Table:** 

Within-day precision:	Standard deviation (rel.)	*s_w_* = 3.3–4.2% or 2.1–3.5%
	Prognostic range	*u* = 9.16–11.7% or 5.83–9.72%
	at a spiked concentration of 5 μg or 150 μg PEN‑OH per litre of urine and n = 5 determinations	
Day-to-day precision:	Standard deviation (rel.)	*s_w_* = 10.7% or 4.1%
	Prognostic range	*u* = 26.2% or 10.0%
	at a spiked concentration of 5 μg or 150 μg PEN‑OH per litre of urine and n = 7 determinations	
Accuracy:	Recovery (rel.)	*r* = 100% or 105%
	at a spiked concentration of 5 μg or 150 μg PEN‑OH per litre of urine and n = 5 determinations	
Limit of detection:	0.5 μg PEN‑OH per litre of urine	
Limit of quantitation:	1.0 μg PEN‑OH per litre of urine	

#### PEN‑COOH

**Table:** 

Within-day precision:	Standard deviation (rel.)	*s_w_* = 3.4–7.5% or 2.1–3.4%
	Prognostic range	*u* = 9.44–20.8% or 5.83–9.44%
	at a spiked concentration of 5 μg or 150 μg PEN‑COOH per litre of urine and n = 5 determinations	
Day-to-day precision:	Standard deviation (rel.)	*s_w_* = 8.7% or 7.4%
	Prognostic range	*u* = 21.3% or 18.1%
	at a spiked concentration of 5 μg or 150 μg PEN‑COOH per litre of urine and n = 7 determinations	
Accuracy:	Recovery (rel.)	*r* = 106% or 107%
	at a spiked concentration of 5 μg or 150 μg PEN‑COOH per litre of urine and n = 5 determinations	
Limit of detection:	0.5 μg PEN‑COOH per litre of urine	
Limit of quantitation:	1.0 μg PEN‑COOH per litre of urine	

## 2 General information on tebuconazole and penconazole

Tebuconazole and penconazole are triazole fungicides that act by inhibiting sterol biosynthesis in fungi (demethyl­ation inhibitor); they are used on several crops, especially vegetables and fruit, including grapes, to control powdery mildew. Tebuconazole and penconazole are present as the active substances in many fungicidal products available on the market, and tebuconazole has been listed among the top ten active fungicidal substances in plant protection (Eurostat 2007). 

In 1994, the Joint Meeting on Pesticide Residues (WHO/FAO) considered triazoles to be safe for humans (WHO and FAO 1994). However, evidence later emerged that high doses of these substances can cause malformations in animals (Giavini and Menegola 2010). In 2008, the European Food Safety Authority (EFSA) proposed classifying tebuconazole as possibly harmful to unborn children (EFSA 2008). Tebuconazole and penconazole have not yet been evaluated by the MAK Commission.

Exposure of workers to tebuconazole and penconazole occurs during the application of related fungicide products, but residents of rural areas in the vicinity of treated areas may also be affected. Furthermore, the general population may be exposed by ingestion of contaminated food products (NCBI 2026). The available data indicate that penconazole and tebuconazole are not only easily absorbed via inhalation, but also via oral and dermal routes (Mercadante et al. 2019; Oerlemans et al. 2019; Sams et al. 2016).

**Fig.1:**
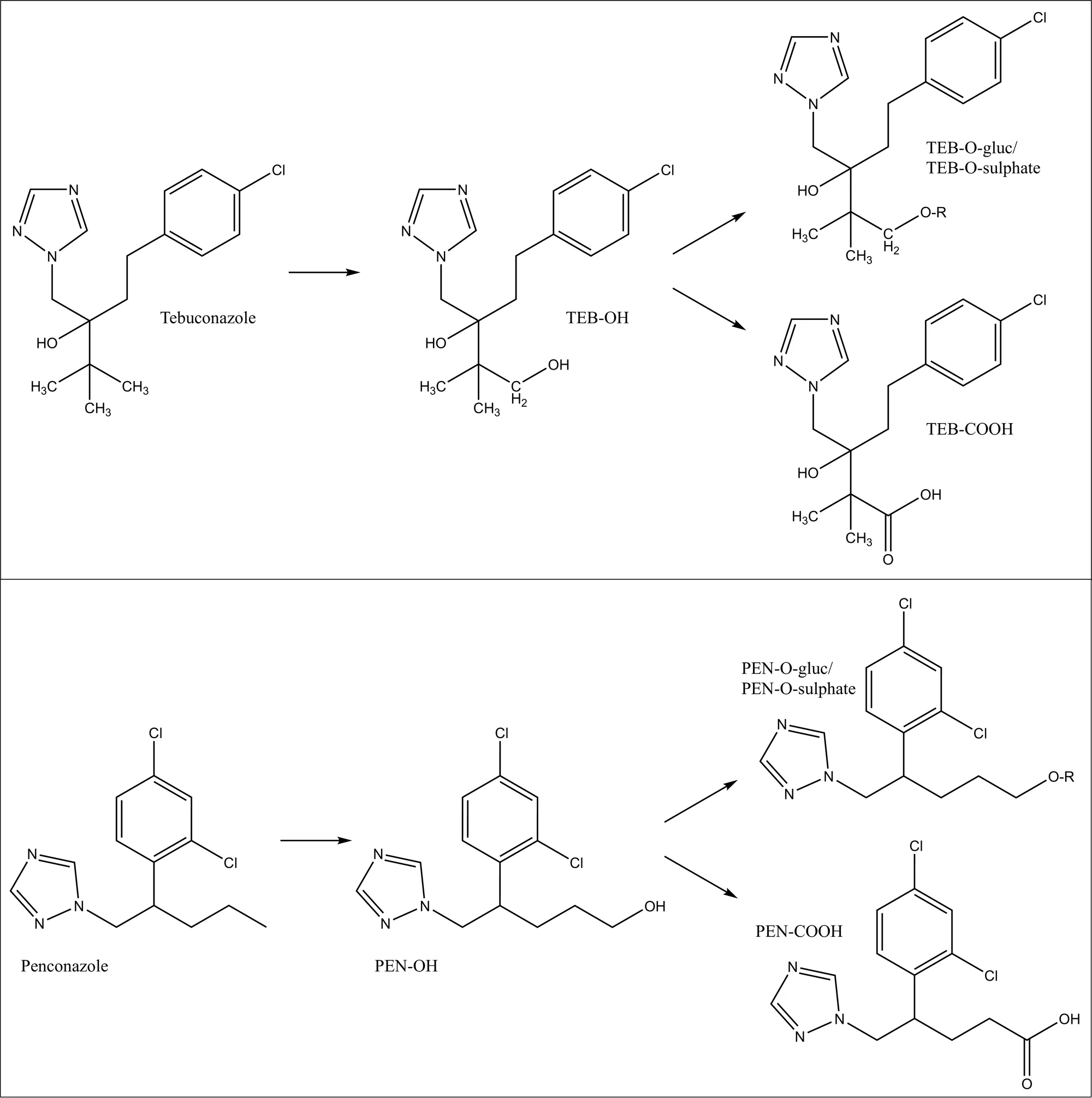
Structural formulas of the fungicides tebuconazole and penconazole as well as their main metabolites

In the human body, orally or dermally absorbed **tebuconazole** is first oxidised to (*RS*)‑5‑(4‑chorophenyl)-2,2‑dimethyl-3‑(1*H*‑1,2,4‑triazol-1‑ylmethyl)-1,3‑pentanediol (TEB‑OH), which is then either glucuronidated, sulfated, or further ­oxidised to form (*RS*)‑5‑(4‑chlorophenyl)-2,2‑dimethyl-3‑(1*H*‑1,2,4‑triazol-1‑ylmethyl)-3‑olpentanoic acid (TEB‑COOH) (WHO and FAO 1994). TEB‑OH conjugates as well as free TEB‑OH and TEB‑COOH are the main metabolites of ­tebuconazole in human urine (Figure 1). Data on the metabolism and toxicokinetics of tebuconazole in humans were first reported by Oerlemans et al. (2019). Following controlled oral and dermal administration of 1.5 mg or 2.5 mg of tebuconazole, a maximum TEB‑OH excretion in the urine after 1.4 h and 20.8 h, respectively, as well as an elimination half-life of 7.8 h and 15.8 h, respectively, were determined. Over 48 h, 38% and 1% of the applied dose, respectively, were excreted as TEB‑OH.

In a field study, urine samples from seven persons occupationally exposed to tebuconazole were collected and analysed for concentrations of TEB‑OH and TEB‑COOH. In five urine samples, the TEB‑OH concentrations without hydrolysis were above the LOQ. After hydrolysis, in four of the five samples, the measured amounts of TEB‑OH were found to be between six and seventy-seven times higher than those of non-hydrolysed samples. In the fifth sample, the TEB‑OH concentration was nearly the same after hydrolysis. The concentration of free TEB‑COOH was higher after hydrolysis as well, whereby the effect was not as pronounced as with TEB‑OH. The results show that the proportion of conjugates may vary significantly between individuals. In the hydrolysed samples, about four times more TEB‑OH could be quantified compared to TEB‑COOH, with concentrations ranging from 9.8 to 473 μg/l or 2.7 to 159 μg/l, respectively (Mercadante et al. 2014).

**Penconazole**, once absorbed into the human body, is also oxidised, whereby 4‑(2,4‑dichlorophenyl-5‑[1,2,4]‑triazol-1‑ylpentanol (PEN‑OH) is first formed. This metabolite is either conjugated to the corresponding glucuronide or sulfate or is further oxidised to the acid 4‑(2,4‑dichlorophenyl)-5‑[1,2,4]‑triazol-1‑ylpentanoic acid (PEN‑COOH) (WHO and FAO 1993). PEN‑OH conjugates and the free PEN‑OH and PEN‑COOH are the most important penconazole metabolites in human urine (Figure 1). In a study by Sams et al. (2016), the metabolism of penconazole after controlled oral administration of 0.03 mg penconazole per kilogram of body weight to three subjects was investigated. The ­maximum excretion rates of both metabolites – PEN‑OH and PEN‑COOH – in urine were reached within the first 2 hours of application. The mean excretion half-lives of PEN‑OH and PEN‑COOH were found to be 3.1 h and 3.7 h, respectively. On average, 47% of the applied dose was excreted over 48 hours, with 83% attributed to PEN‑OH and 17% to PEN‑COOH. 

In a study on workers applying penconazole in wine-growing regions, the internal exposure correlated well with their current dermal exposure, which means that penconazole can likewise be assumed to have absorption and toxico­kinetics comparable to tebuconazole after dermal exposure (Mercadante et al. 2019).

To estimate occupational exposure to penconazole, urine samples from 22 occupationally exposed workers were collected and analysed using the method hereby presented. The concentrations of penconazole metabolites in the individual urines varied widely, whereby this variability was considerably reduced by hydrolysis of the samples (Mercadante et al. 2016). The workers were examined after penconazole application as well as after re-entry into penconazole-treated areas. The spray liquid was prepared and applied on up to four consecutive workdays (n = 42), and the treated areas were re-entered twelve times. Urine samples were collected before the first application, during preparation, during application, and up to 48 h after the last shift. The PEN‑OH concentrations lied significantly above those of PEN‑COOH, and, 24 h after application, ranged from 15.6 to 27.6 μg PEN‑OH/l or 2.5 to 10.2 μg/l PEN‑COOH/l (Mercadante et al. 2019).

Table 1 provides representative background concentrations of tebuconazole and TEB‑OH in urine from the general population. Table 2 gives representative concentrations of tebuconazole, TEB‑OH, and TEB‑COOH in urine from occupationally exposed persons. In Table 3, representative background concentrations of PEN‑COOH in urine from the general population are presented, and Table 4 shows representative concentrations of PEN‑OH and PEN‑COOH in urine from occupationally exposed persons.

**Tab.1:** Tebuconazole and its metabolites in urine from the non-occupationally exposed general population

Study collective (country; n; sample)	Analytes	DF [%]	QF [%]	LOD [μg/l]	LOQ [μg/l]	GM [μg/l]	Median [μg/l]	P95 [μg/l]	Range [μg/l]	Analytical method	References
Pregnant women (Argentina; 89; first-morning void)	TEB	12.4	8.9	0.3	1.0	n. s.	n. s.	n. s.	0.1–0.3	GC-MS/MS	Racca et al. 2025
Children aged 7–9 years (Poland; 399; spot urine)	TEB	12	n. s.	0.041	n. s.	< LOQ	< LOQ	0.056^a)^	< LOQ–0.076^a)^	UPLC-MS/MS	Bustamante et al. 2025
	TEB‑OH	96	n. s.	0.064	n. s.	0.3^a)^	0.35^a)^	1.8^a)^	< LOQ–19^a)^		
Pregnant women (Germany; 587; first-morning void)	TEB	1	n. s.	n. s.	n. s.	n. s.	0.3	n. s.	n. s.	HPLC-HRMS	Huber et al. 2024
	TEB-(OH)_2_	57	n. s.	n. s.	n. s.	n. s.	n. s.	n. s.	n. s.		
Adults (EU; 110; first-morning void)	TEB‑OH	n. s.	98.2	0.02	0.05	n. s.	0.47	1.74	n. s.	HPLC-MS/MS	Šulc et al. 2022
School-age children (EU; 110; first-morning void)	TEB‑OH	n. s.	99.1	0.02	0.05	n. s.	0.44	1.77	n. s.		

DF: detection frequency (measured values above LOD); GM: geometric mean; LOD: limit of detection; LOQ: limit of quantitation; n: sample size; n. s.: not stated; P95: 95th percentile; QF: quantitation frequency (measured values above LOQ); TEB: tebuconazole

a) calculated using LOQ/2 for values below the LOQ

**Tab.2:** Tebuconazole and its metabolites in urine from exposed workers

Study collective (country; n; sample)	Analytes	DF [%]	QF [%]	LOD [μg/l]	LOQ [μg/l]	GM (GSD) [μg/l]	Median [μg/l]	P95 [μg/l]	Range [μg/l]	Analytical method	References
Farm workers (Mexico; 105; spot urine)	TEB‑OH	76	n. s.	0.10	n. s.	0.36	0.34	n. s.	< LOD–4.17	LC-MS/MS	Alcalá et al. 2024
Vineyard workers (Italy; 3; spot urine 24 h after 2^nd^ work shift)	TEB	n. s.	n. s.	0.5	1.5	n. s.	4.6	n. s.	3.7–6.0	LC-MS/MS	Fustinoni et al. 2014
	TEB‑OH	n. s.	n. s.	0.2	0.3	n. s.	249.0	n. s.	58.3–383.6		
	TEB‑COOH	n. s.	n. s.	0.1	0.3	n. s.	51.1	n. s.	17.3–100.4		
Vineyard workers (Italy; 7; spot urine 24 h after application)	TEB‑OH	n. s.	n. s.	0.2	0.3	125.6±175.3^a)^	n. s.	n. s.	n. s.	LC-MS/MS	Mercadante et al. 2014
	TEB‑COOH	n. s.	n. s.	0.1	0.3	34.5±56.7^a)^	n. s.	n. s.	n. s.		
Farm workers (Costa Rica; 299; spot urine)	TEB‑OH	95.6–97.1	n. s.	0.05	n. s.	0.91 (3.44)	0.88	n. s.	< LOD–35.54	LC-MS/MS	Rodríguez-Zamora et al. 2024

DF: detection frequency (measured values above LOD); GM: geometric mean; GSD: geometric standard deviation; LOD: limit of detection; LOQ: limit of quantitation; n: sample size; n. s.: not stated; P95: 95th percentile; QF: quantitation frequency (measured values above LOQ); TEB: tebuconazole

a) arithmetic mean ± standard deviation

**Tab.3:** PEN-COOH in urine from the non-occupationally exposed general population

Study collective (country; n; sample)	Analytes	DF [%]	QF [%]	LOD [μg/l]	LOQ [μg/l]	GM [μg/l]	Median[μg/l]	P95 [μg/l]	Range [μg/l]	Analytical method	References
Rural residents (United Kingdom; 483; spot urine outside of spray season)	PEN-COOH	12	n. s.	0.25	n. s.	n. s.	n. s.	0.22	< LOD–1.73	LC-MS/MS	Sams et al. 2016
Rural residents (United Kingdom; 556; spot urine from within spray season)	PEN-COOH	10	n. s.	0.25	n. s.	n. s.	n. s.	0.29	< LOD–1.19		

DF: detection frequency (measured values above LOD); GM: geometric mean; LOD: limit of detection; LOQ: limit of quantitation; n: sample size; n. s.: not stated; PEN: penconazole; P95: 95th percentile; QF: quantitation frequency (measured values above LOQ)

**Tab.4:** Penconazole and its metabolites in urine from exposed workers

Study collective (country; n; sample)	Analytes	DF [%]	QF [%]	LOD [μg/l]	LOQ [μg/l]	GM (GSD) [μg/l]	Median [μg/l]	P95 [μg/l]	Range [μg/l]	Analytical method	References
Agricultural workers (Italy; 5; spot urine)	PEN	n. s.	n. s.	n. s.	2.0	n. s.	< LOQ	n. s.	< LOQ–3.1	LC-MS/MS	Mercadante et al. 2016
	PEN-OH	n. s.	n. s.	n. s.	1.0	n. s.	290.0	n. s.	230.1–464.3		
	PEN-COOH	n. s.	n. s.	n. s.	1.0	n. s.	7.9	n. s.	5.2–16.7		
Vineyard workers (Italy; 22; 24 h-pooled urine 25–48 h post shift)	PEN-OH	n. s.	n. s.	n. s.	1.0	n. s.	9.4	n. s.	1.2–237.0	LC-MS/MS	Mercadante et al. 2019
	PEN-COOH	n. s.	n. s.	n. s.	1.0	n. s.	1.8	n. s.	< 1.0–50.9		
Farm workers (Costa Rica; 299; spot urine)	PEN-OH	38.9–44.0	n. s.	0.05	n. s.	0.06 (2.09)	0.06	n. s.	< LOD–0.63	LC-MS/MS	Rodríguez-Zamora et al. 2024

DF: detection frequency (measured values above LOD); GM: geometric mean; GSD: geometric standard deviation; LOD: limit of detection; LOQ: limit of quantitation; n: sample size; n. s.: not stated; PEN: penconazole; P95: 95th percentile; QF: quantitation frequency (measured values above LOQ)

## 3 General principles

The analytical method described herein permits the selective and sensitive determination of TEB‑OH, TEB‑COOH, PEN‑OH, and PEN‑COOH in urine. After adding the labelled ISTDs (tebuconazole‑D_6_ and penconazole‑D_7_), the samples are enzymatically hydrolysed to release the analytes from the glucuronide and sulfate conjugates. After online purification, the analytes are separated by liquid chromatography and quantified using tandem mass spectrometry. Calibration is performed using calibration standards prepared in pooled urine and processed analogously to the samples to be analysed.

## 4 Equipment, chemicals, and solutions

### 4.1 Equipment

- LC system consisting of a TurboFlow system with two gradient pumps, an eluent degasser, and an autosampler (e.g. Thermo Fisher Scientific S.p.A., Rodano, Italy)
- Tandem mass spectrometer (e.g. TSQ Quantum Access, Thermo Fisher Scientific S.p.A., Rodano, Italy)
- TurboFlow^TM^ column (e.g. No. CH-953288, TurboFlow^TM^ Cyclone column, length: 50 mm, inner diameter: 0.5 mm, Thermo Fisher Scientific S.p.A., Rodano, Italy)
- Analytical LC column (e.g. No. 25403-052130, Hypersil Gold PFP, length: 50 mm, inner diameter: 2.1 mm, particle size: 3 μm, Thermo Fisher Scientific S.p.A., Rodano, Italy)
- Laboratory shaker (e.g. VIBROMIX, Domel Holding, d.o.o., Železniki, Slovenia)
- Analytical balance (e.g. Mettler-Toledo GmbH, Gießen, Germany)
- Drying oven (e.g. Model STZ 5.4, Ettore Pasquali S.r.l., Milano, Italy)
- Various volumetric flasks, glass beakers, and measuring cylinders (e.g. Schott AG, Mainz, Germany)
- Various pipettes and Multipettes^®^ with matching pipette tips (e.g. Gilson International BV Deutschland, Berlin, Germany, or Eppendorf AG, Hamburg, Germany)
- 2‑ml crimp-neck vials with crimp caps (e.g. Agilent Technologies Germany GmbH & Co. KG, Waldbronn, Germany)
- Urine-collection cups (e.g. SARSTEDT AG & Co. KG, Nümbrecht, Germany)

### 4.2 Chemicals

Unless otherwise specified, all chemicals must be a minimum of *pro analysi* grade.

- Acetic acid (100%, glacial acetic acid) (e.g. No. 1.00063, Merck KGaA, Darmstadt, Germany)
- Acetone, EMSURE^®^ (e.g. No. 1.00014, Merck KGaA, Darmstadt, Germany)
- Acetonitrile, SupraSolv^®^ (e.g. No. 100017, Merck KGaA, Darmstadt, Germany)
- Ammonium hydroxide solution, 28–30% NH_3_ (e.g. No. 221228, Merck KGaA, Darmstadt, Germany)
- *β*‑Glucuronidase from *Helix* *pomatia*, type H‑2 (≥ 100 000 U/ml glucuronidase activity and ≤ 7500 U/ml sulfatase activity) (e.g. No. G7017, Merck KGaA, Darmstadt, Germany)
- Methanol, LiChrosolv^®^ (e.g. No. 1.06007, Merck KGaA, Darmstadt, Germany)
- 2-Propanol, LiChrosolv^®^ (e.g. No. 1.02781, Merck KGaA, Darmstadt, Germany)
- Sodium acetate trihydrate, EMSURE^®^ (e.g. No. 1.06267, Merck KGaA, Darmstadt, Germany)
- Ultra-pure water (e.g. Milli‑Q^®^ ultra-pure water system, Merck KGaA, Darmstadt, Germany)
- Tebuconazole‑D_6_, 100 μg/ml in acetone (e.g. No. DRE‑XA17178710AC, Dr. Ehrenstorfer, LGC Standards GmbH, Wesel, Germany)
- (*RS*)-5‑(4‑Chlorophenyl)-2,2‑dimethyl-3‑(1*H*‑1,2,4‑triazol-1‑ylmethyl)-1,3‑pentanediol (TEB‑OH), technical grade, 97.5% (kindly donated by Bayer CropScience Deutschland GmbH, Monheim, Germany)
- (*RS*)-5‑(4‑Chlorophenyl)-2,2‑dimethyl-3‑(1*H*‑1,2,4‑triazol-1‑ylmethyl)-3‑olpentanoic acid (TEB‑COOH), technical grade, 97.5% (kindly donated by Bayer CropScience Deutschland GmbH, Monheim, Germany)
- Penconazole‑D_7_ (e.g. No. AABH9A22149F, Merck KGaA, Darmstadt, Germany)
- 4‑(2,4‑Dichlorophenyl)-5‑[1,2,4]‑triazol-1‑ylpentanol (PEN-OH), technical grade, 98% (kindly donated by Syngenta Group, Cambridge, United Kingdom)
- 4‑(2,4‑Dichlorophenyl)-5‑[1,2,4]-triazol-1‑ylpentanoic acid (PEN-COOH), technical grade, 98% (kindly donated by Syngenta Group, Cambridge, United Kingdom)

### 4.3 Solutions

- Eluent C (acetonitrile : 2-propanol : acetone (45 ∶ 45 ∶ 10; v/v/v))
  In a 500‑ml volumetric flask, 225 ml of acetonitrile, 225 ml of 2-propanol, and 50 ml of acetone are added and mixed by shaking.
- Eluent D (acetonitrile : ammonium hydroxide (99.97 : 0.03; v/v))
  A little acetonitrile is placed in a 1‑l volumetric flask and 1 ml of the 30% ammonium hydroxide solution is added. The flask is then made up to the mark with acetonitrile.

The solutions are stable at 25 °C for at least six months.

- Eluent F (acetic acid (0.5%))
  A little ultra-pure water is placed in a 1‑l volumetric flask and 5 ml of glacial acetic acid are added. The flask is then made up to the mark with ultra-pure water.

The solution is stable at 25 °C for at least six months.

- Acetic acid (0.5 mol/l)
  A little ultra-pure water is placed in a 50-ml volumetric flask and 1.43 ml of glacial acetic acid are added by pipetting. The flask is then made up to the mark with ultra-pure water.

The solution is stable at 4 °C for at least three months.

- Sodium acetate solution (0.5 mol/l)
  Exactly 3.4 g of sodium acetate trihydrate are weighed into a 50-ml volumetric flask. The flask is then made up to the mark with ultra-pure water.

The solution is stable at 4 °C for at least three months.

- Sodium acetate buffer (pH 5.5)
  In a volumetric flask, 25 ml of the sodium acetate trihydrate solution (0.5 mol/l) are mixed with 13.75 ml of the acetic acid solution (0.5 mol/l).

The sodium acetate buffer solution is stable at 4 °C for at least three months.

- *β‑*Glucuronidase solution
  In a volumetric flask, 1 ml of *β*‑glucuronidase is mixed with 27 ml of the sodium acetate buffer.

The *β‑*glucuronidase solution is stable at 4 °C for at least six months.

### 4.4 Internal standards (ISTDs)

- Penconazole-D_7_ stock solution (100 mg/l)
  About 1 mg of penconazole‑D_7_ is weighed into a 10-ml volumetric flask and dissolved in methanol. The volumetric flask is then made up to the mark with methanol.
- ISTD spiking solution (10 mg/l)
  1 ml of the tebuconazole‑D_6_ standard and 1 ml of the penconazole‑D_7_ stock solution are pipetted into a 10‑ml volumetric flask. The flask is then made up to the mark with methanol.

The penconazole‑D_7_ stock solution and the ISTD spiking solution are stored in the dark at −20 °C; under these conditions, the solutions are stable for at least six months.

### 4.5 Calibration standards

- Stock solutions I (1000 mg/l)
  Exactly 10 mg each of TEB‑OH, TEB‑COOH, PEN‑OH, and PEN‑COOH are weighed into separate 10‑ml volumetric flasks and dissolved in a little methanol. The flasks are then made up to the mark with methanol.
- Stock solutions II (100 mg/l)
  Each Stock solution I is individually diluted by pipetting 1 ml of the corresponding solution into a 10‑ml volumetric flask, which is then made up to the mark with methanol.
- Spiking solution I (10 mg/l TEB‑OH, 4 mg/l TEB‑COOH, 10 mg/l PEN‑OH, 10 mg/l PEN‑COOH)
  1000 μl of Stock solution II of TEB‑OH, 400 μl of Stock solution II of TEB‑COOH, 1000 μl of Stock solution II of PEN‑OH, and 1000 μl of Stock solution II of PEN‑COOH are pipetted into a 10‑ml volumetric flask, which is then made up to the mark with methanol.
- Spiking solution II (1 mg/l TEB‑OH, 0.4 mg/l TEB‑COOH, 1 mg/l PEN‑OH, 1 mg/l PEN‑COOH)
  1 ml of Spiking solution I is pipetted into a 10‑ml volumetric flask, which is then made up to the mark with methanol.
- Spiking solution III (1 mg/l TEB‑OH, 0.4 mg/l TEB‑COOH, 0.1 mg/l PEN‑OH, 0.1 mg/l PEN‑COOH)
  100 μl of Stock solution II for TEB‑OH, 40 μl of Stock solution II for TEB‑COOH, 10 μl of Stock solution II for PEN‑OH, and 10 μl of Stock solution II for PEN‑COOH are pipetted into a 10‑ml volumetric flask, which is then made up to the mark with methanol.

The stock and spiking solutions are stored in the dark at −20 °C; under these conditions, the solutions are stable for at least six months.

The calibration standards are prepared in pooled urine. For the pooled urine, spot-urine samples from individuals not occupationally exposed to tebuconazole and penconazole are collected in suitable containers, mixed, and stored at −20 °C until preparation of the standards and control materials.

According to the pipetting scheme given in Table 5, calibration standards are prepared by spiking the pooled urine in the following concentration ranges: 10–500 μg TEB‑OH per litre of urine, 4–200 μg TEB‑COOH per litre of urine, 1–500 μg PEN‑OH per litre of urine, and 1–500 μg PEN‑COOH per litre of urine. The unspiked pooled urine is additionally included as a blank.

**Tab.5:** Pipetting scheme for the preparation of calibration standards for the determination of TEB‑OH, TEB‑COOH, PEN‑OH, and PEN‑COOH in urine

Calibration standard	Spiking solution	Spiking solution [μl]	Pooled urine [μl]	Analyte concentration [μg/l]			
				TEB‑OH	TEB‑COOH	PEN‑OH	PEN‑COOH
0	–	–	1000	–	–	–	–
1	III	10	990	10	4	1	1
2	II	25	975	25	10	25	25
3	II	50	950	50	20	50	50
4	I	10	990	100	40	100	100
5	I	20	980	200	80	200	200
6	I	50	950	500	200	500	500

## 5 Specimen collection and sample preparation

### 5.1 Specimen collection

The urine samples are collected in suitable urine-collection cups and stored in the dark at −20 °C until analysis.

### 5.2 Sample preparation

Prior to analysis, the urine samples are thawed at room temperature and mixed thoroughly. For sample preparation, 1 ml of the urine sample is pipetted into a 2‑ml crimp-neck vial and 5 μl of the ISTD spiking solution as well as 100 μl of the *β*‑glucuronidase solution are added. The vial is sealed and the sample is thoroughly mixed on a vortex mixer. Subsequently, the sample is incubated overnight (16 h) at 37 °C and, after cooling to room temperature, directly injected into the LC‑MS/MS system.

## 6 Operational parameters

Analysis is carried out using a device configuration comprised of a TurboFlow-LC system and a tandem mass spectrometer (TurboFlow‑LC‑MS/MS). Turbulent-flow chromatography is a relatively new technique able to extract and purify biological sample materials online, thereby – similar to an online SPE – simplifying sample preparation and saving time. The corresponding TurboFlow^TM^ columns can be used in liquid-chromatographic instrumentation from all manufacturers, but it is important to note that the only instruments on the market designed to take full advantage of TurboFlow^TM^ columns are the Transcend TLX systems sold by Thermo Fisher Scientific.

The TurboFlow method described involves the application of a TurboFlow^TM^ Cyclone column based on a styrene/­di­vinylbenzene‑copolymer, suitable for non-polar analytes in complex matrices (pH values in the range of 1–13).

### 6.1 Liquid chromatography

The TurboFlow system uses a quaternary pump (loading pump) to transport the sample onto the TurboFlow^TM^ ­column as well as for extraction/purification of the sample. A second, binary pump (elution pump) serves to separate the extract via a conventional analytical column.

**Table:** 

Enrichment column (TurboFlow^TM^ column):	TFLC Cyclone column, 50 × 0.5 mm (on a styrene/divinylbenzene-copolymer basis)
Analytical column:	Hypersil Gold PFP, 3 μm, 50 × 2.1 mm
Mobile phases of quaternary pump P1:	Eluent A: ultra-pure water
	Eluent B: acetonitrile
	Eluent C: acetonitrile : 2-propanol : acetone (45 ∶ 45 ∶ 10; v/v/v)
	Eluent D: acetonitrile : ammonium hydroxide (99.97 : 0.03; v/v)
Mobile phases of binary pump P2:	Eluent E: methanol
	Eluent F: 0.5% acetic acid
Flow rate of quaternary pump P1:	see Table 6
Flow rate of binary pump P2:	0.7 ml/min
Column temperature:	Room temperature
Injection volume:	50 μl
Gradient programme:	see Table 6
Valve-switching programme:	see Table 7

**Tab.6:** Gradient programme for the determination of TEB‑OH, TEB‑COOH, PEN‑OH, and PEN‑COOH in urine

Time [min]	Sample cleanup with TurboFlow^TM^ column – quaternary pump P1							Chromatographic separation – binary pump P2		
	Flow rate [ml/min]	Eluent A [%]	Eluent B [%]	Eluent C [%]	Eluent D [%]	T-piece	Loop	Gradient	Eluent E [%]	Eluent F [%]
0	1.0	100	0	0	0	–	out	step	0	100
1.0	0.15	100	0	0	0	–	out	step	0	100
1.08	0.15	100	0	0	0	T	in	step	0	100
3.75	1.0	0	0	100	0	–	in	linear	70	30
4.92	1.0	0	0	100	0	–	in	step	70	30
12.0	1.0	0	0	0	100	–	in	step	100	0
13.0	1.0	30	70	0	0	–	in	linear	0	100
14.0	1.0	100	0	0	0	–	out	step	0	100
16.0	1.0	100	0	0	0	–	out	step	0	100

The TurboFlow-chromatography mode used for this method is named “Focus Mode”, as the analytes are focused in the head of the analytical column prior to chromatographic separation. This Focus Mode consists of six steps: loading, sample washing, sample transfer, system washing, filling the elution loop, and reconditioning.

**Fig.2:**
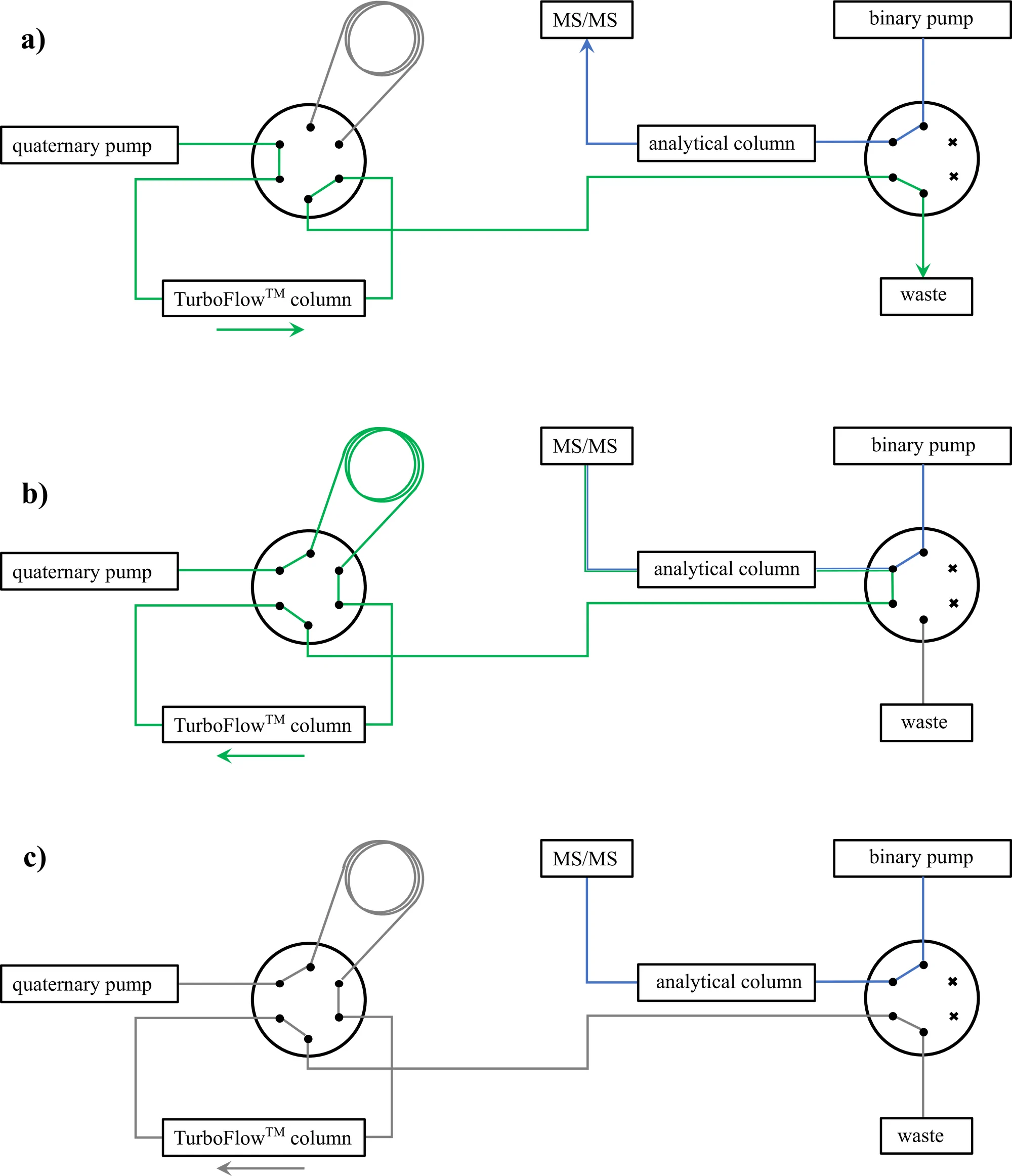
Flow chart of the LC TurboFlow system: a) loading and purification, b) transfer onto analytical column in backflush mode, ­­c) ­chromatographic separation followed by equilibration

In the loading step, samples are injected into the TurboFlow^TM^ column at high flow rates so that macromolecules of the matrix, salts, and ionic compounds, which are not retained by the column, flow to waste. Once loaded, the sample is cleaned of potential contaminants by the mobile phase. During these two steps, the analytical column connected to the detector is flushed with Eluent F of the binary pump (Figure 2, a).

For the following transfer step, valves A and B are switched. The solvent mixture from the loop is pushed into the TurboFlow^TM^ column by the mobile phase of the quaternary pump in backflush mode. The analytes are eluted off and transferred onto the analytical column (Figure 2, b). In this step, the solvent mixture of the loop is diluted by the mobile phase from the binary pump (via a T-piece in valve B) before reaching the analytical column in order to reduce solvent strength and to focus the target analytes in the head of the analytical column.

When sample transfer is complete, valve B is switched, and the loading and eluting flows are again separated. While the analytes are separated on the analytical column and eluted to the detector, the loop and the TurboFlow^TM^ column are rinsed to avoid carryover (Figure 2, c). Subsequently, the loop is again filled with the eluent for the transfer step to be used for the next sample (Figure 2, c). In the final step, the loop is isolated, and the columns are re-equilibrated to the conditions of the loading step (Figure 2, a).

**Tab.7:** Valve-switching programme

Time [min]	Valve switching	Description
0.00–1.08	A	Enrichment and purification of the analytes on the TurboFlow^TM^ column
1.08–3.75	A, B	Transfer of the analytes onto the analytical column in backflush mode
3.75–16.0	B	Chromatographic separation of the analytes, washing, and equilibration of the enrichment column

### 6.2 Tandem mass spectrometry

**Table:** 

Ionisation mode:	Positive heated electrospray ionisation (HESI+)
Source temperature:	199 °C
Desolvation temperature:	211 °C
Detection mode:	Multiple Reaction Monitoring (MRM)
Sheath-gas pressure:	25 (arbitrary scale)
Auxiliary-gas pressure:	15 (arbitrary scale)
Spray voltage:	4500 V
Tube-lens offset:	104 V

All ion-source settings and MRM parameters are instrument-specific and must be adjusted individually by the user. The parameters specified in this section are therefore intended only as a point of reference. All other parameters must be optimised in accordance with manufacturer specifications.

## 7 Analytical determination

50 μl of the sample prepared as described in Section 5 are injected into the LC‑MS/MS system. Identification of the analytes is based on their retention times and specific mass transitions. The retention times of the analytes and ISTDs as well as the ion traces used are presented in Table 8. In addition to the calibration standards, several reagent blank samples (ultra-pure water instead of a urine sample) and two quality-control samples are included in each analytical run (see Section 10).

**Tab.8:** Retention times, ion transitions, and collision energy for the determination of TEB‑OH, TEB‑COOH, PEN‑OH, and PEN‑COOH in urine

Analyte or ISTD	Retention time [min]	Ion trace (*m/z*)		Collision energy [eV]
		Q1	Q3	
TEB‑OH	5.25	324	70^a)^	21
		324	151	30
TEB‑COOH	5.20	338	70^a)^	27
		338	163	28
Tebuconazole‑D_6_	5.38	314	72^a)^	22
		314	125	25
		314	154	30
PEN‑OH	5.07	300	70^a)^	15
		300	159	27
PEN‑COOH	5.01	314	70^a)^	13
		314	159	29
Penconazole‑D_7_	5.50	291	70^a)^	15
		291	159	33

a) quantifier

**Fig.3:**
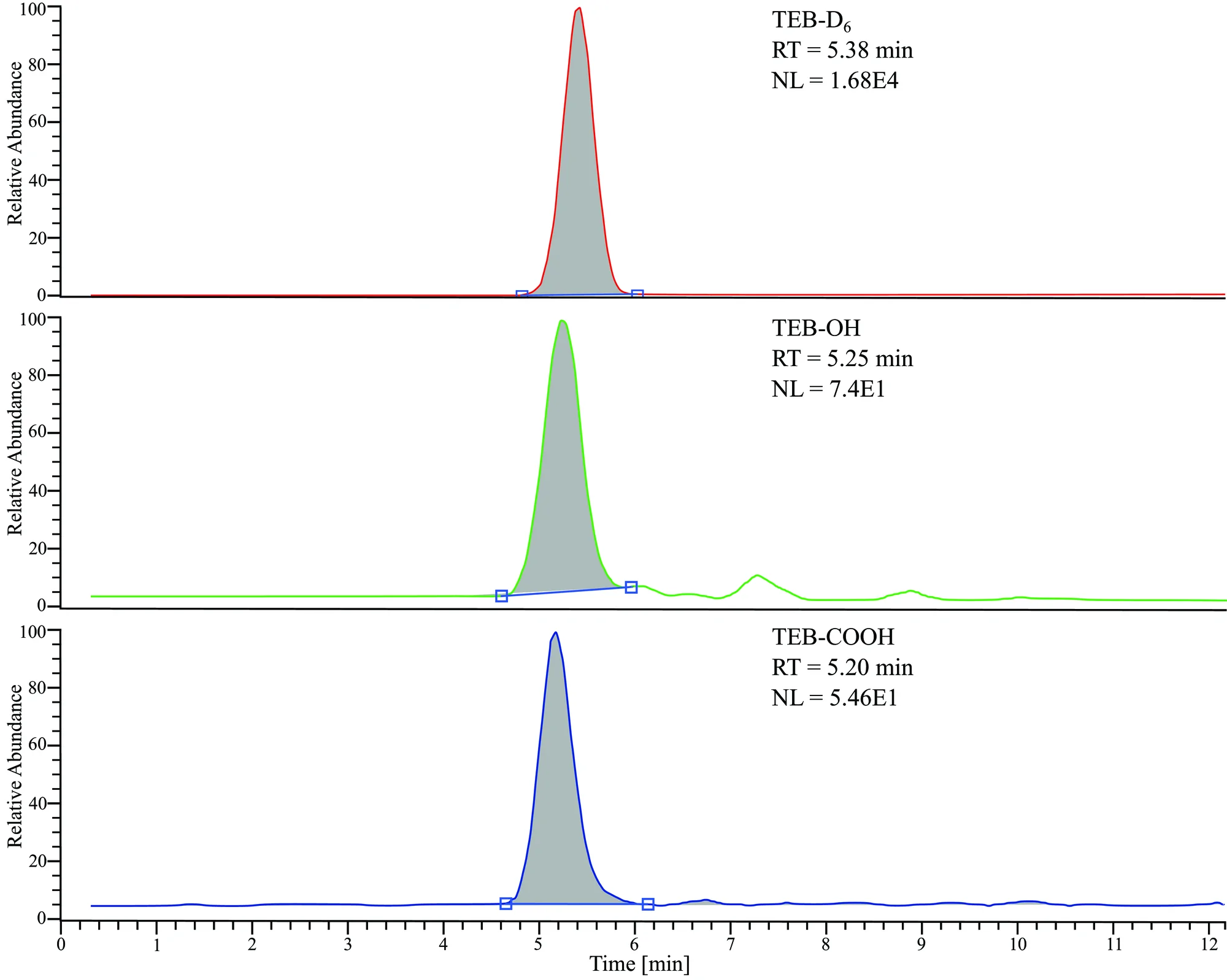
Chromatograms of a urine sample spiked with TEB‑OH and TEB‑COOH at the limit of quantitation with retention time (RT) and normalisation level (NL) indicated

**Fig.4:**
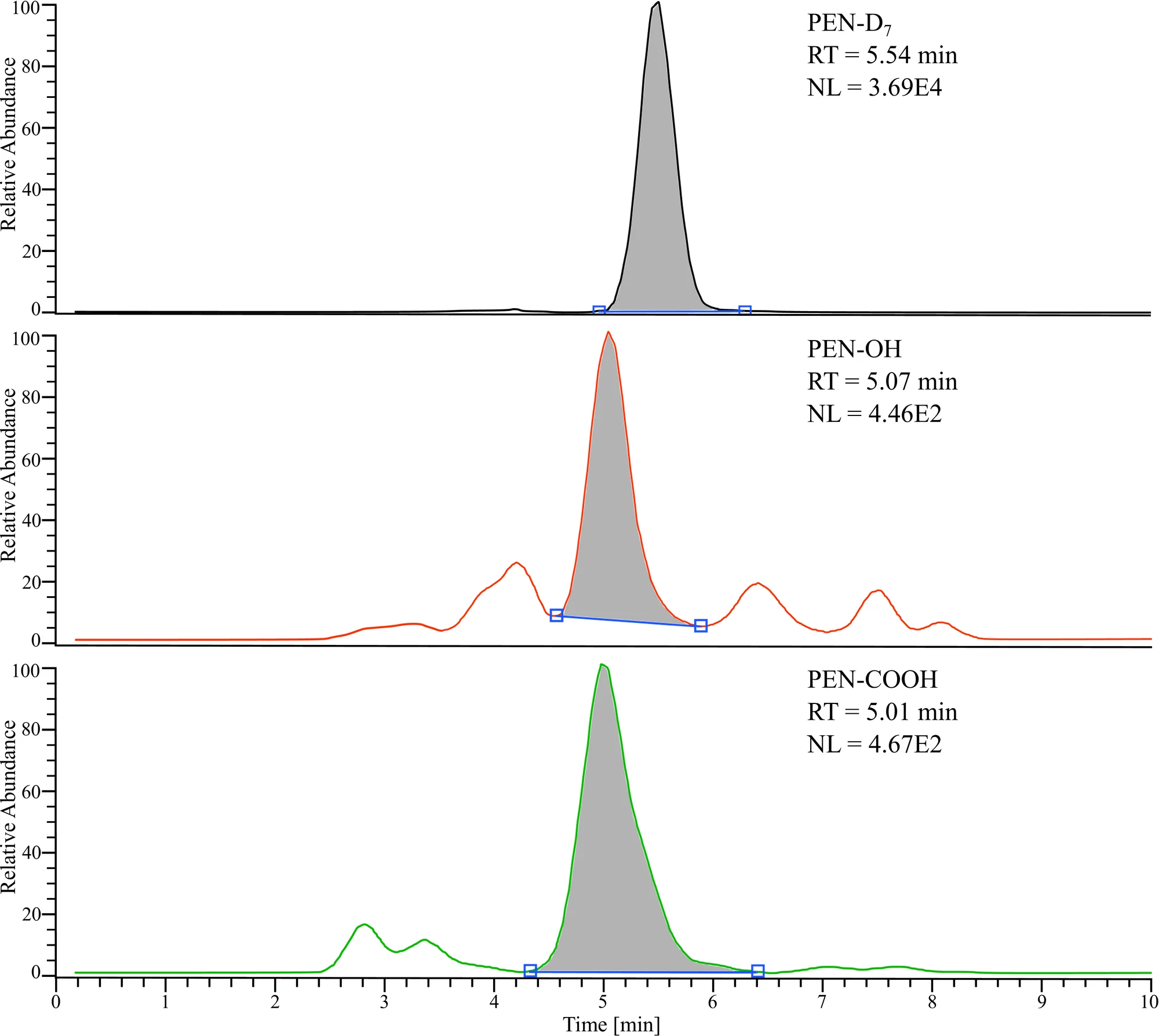
Chromatograms of a urine sample spiked with PEN-OH and PEN-COOH at the limit of quantitation with retention time (RT) and normalisation level (NL) indicated

The retention times given in Table 8 are intended only as cursory guidance and may differ based on the LC instrument setup used. Users must ensure proper separation performance of the analytical column used and the resulting retention behaviour of the analytes. Figures 3 and 4 show exemplary chromatograms of a urine sample spiked with the analytes at the limit of quantitation.

## 8 Calibration

**Fig.5:**
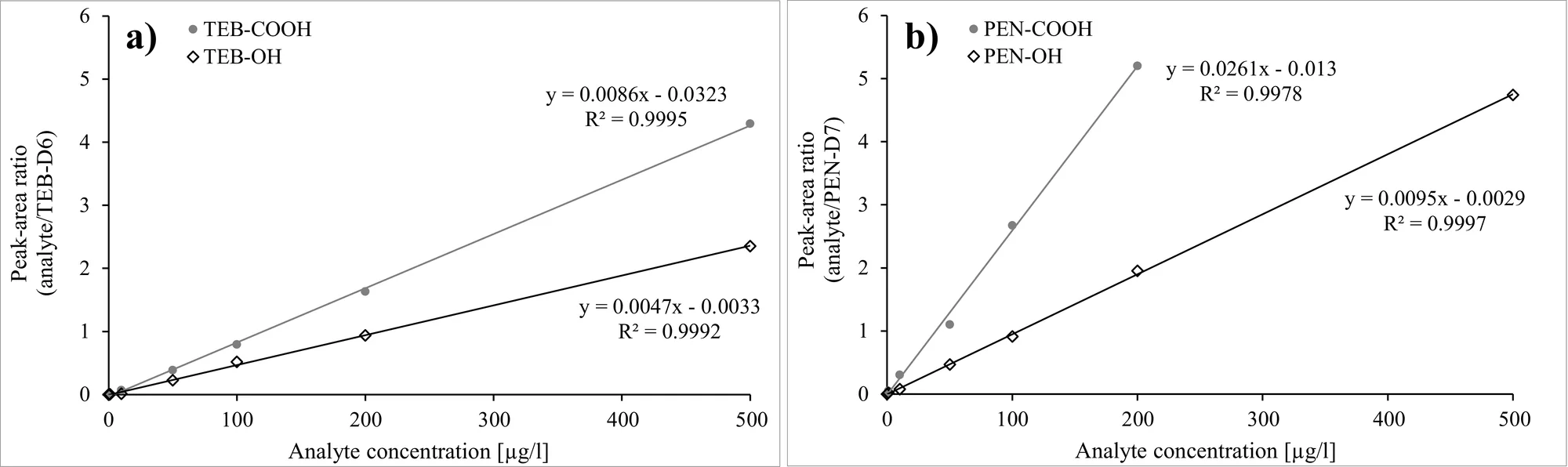
Calibration curves for the determination of TEB‑OH and TEB‑COOH (a) as well as of PEN-OH and PEN-COOH (b) in urine

The calibration standards are prepared as described in Section 4.5, processed in the same way as the samples (see Section 5), and analysed by LC‑MS/MS (see Section 6). Calibration curves are obtained by plotting the peak-area ratios of the analytes and the corresponding ISTDs against the concentrations of the calibration standards. Under the analytical conditions described, the calibration curves are linear in the concentration ranges of 10–500 μg/l for TEB‑OH, 4–200 μg/l for TEB‑COOH, 1–500 μg/l for PEN‑OH as well as 1–500 μg/l for PEN‑COOH. Figure 5 shows examples of calibration curves for the determination of tebuconazole and penconazole metabolites in urine.

After the calibration standards, a reagent blank is measured as part of each analytical run to monitor any potential analyte carryover (cf. Section 11.4).

## 9 Calculation of the analytical results

The analyte concentrations in the urine samples are calculated using the calibration function of the corresponding analytical run (Section 8). The peak area of the respective analyte is divided by the peak area of its ISTD. The ratio thus obtained is entered into the calibration function, yielding the respective analyte concentration in μg/l.

## 10 Standardisation and quality control

Quality control of the analytical results was carried out as stipulated in the guidelines of the U.S. Food and Drug Administration (FDA) (FDA 2018).

To check precision, at least two quality-control samples with known analyte concentrations are included in each analytical run. As material for quality control is not commercially available, it must be prepared in the in-house laboratory. To this end, urine from individuals not occupationally exposed to tebuconazole or penconazole is collected and spiked with the standard analyte solutions, so that the resulting analyte concentrations in the control materials are within the relevant range.

The developers of the method used the following analyte concentrations for quality control: 20 μg TEB‑OH/l, 8 μg TEB‑COOH/l, 5 μg PEN‑OH/l, and 5 μg PEN‑COOH/l for Q_low_; 150 μg TEB‑OH/l, 60 μg TEB‑COOH/l, 150 μg PEN‑OH/l, and 150 μg PEN‑COOH/l for Q_high_.

A reagent blank should be analysed after Q_high_ to identify any potential carryover effects (cf. Section 11.4).

## 11 Evaluation of the method

The reliability of this method was confirmed by comprehensive validation as well as by replication and verification in a second, independent laboratory. For method verification, a UPLC system from Waters GmbH (Eschborn, Germany) in combination with a TurboFlow^TM^ column from Thermo Fisher Scientific GmbH (Dreieich, Germany) was used. The operational parameters applied during method verification are given in the Appendix.

### 11.1 Precision

Precision data were determined by processing and analysing the quality-control materials five times in parallel over seven days. Table 9 shows the ranges of the within-day precision data determined for each analyte over the seven-day measurement period.

**Tab.9:** Within-day precision for the determination of TEB‑OH, TEB‑COOH, PEN‑OH, and PEN‑COOH in urine (n = 5)

Analyte	Spiked concentration [μg/l]	Standard deviation (rel.) *s_w_* [%]	Prognostic range *u* [%]
TEB‑OH	20	0.1–1.7	0.28–4.72
	150	0.1–1.4	0.28–3.89
TEB‑COOH	8	0.4–3.1	1.11–8.61
	60	0.1–0.9	0.28–2.50
PEN‑OH	5	3.3–4.2	9.16–11.7
	150	2.1–3.5	5.83–9.72
PEN‑COOH	5	3.4–7.5	9.44–20.8
	150	2.1–3.4	5.83–9.44

To determine day-to-day precision, the relative standard deviations were calculated from the respective mean values over the seven-day measurement period. The precision data thus obtained are presented in Table 10.

**Tab.10:** Day-to-day precision for the determination of TEB‑OH, TEB‑COOH, PEN‑OH, and PEN‑COOH in urine (n = 7)

Analyte	Spiked concentration [μg/l]	Standard deviation (rel.) *s_w_* [%]	Prognostic range *u* [%]
TEB‑OH	20	1.0	2.45
	150	0.8	1.96
TEB‑COOH	8	1.6	3.92
	60	1.4	3.43
PEN‑OH	5	10.7	26.2
	150	4.1	10.0
PEN‑COOH	5	8.7	21.3
	150	7.4	18.1

### 11.2 Accuracy

The relative recoveries of the individual analytes were determined based on the within-day precision data. The data thus calculated are given in Table 11.

**Tab.11:** Mean relative recoveries for the determination of TEB‑OH, TEB‑COOH, PEN‑OH, and PEN‑COOH in urine (n = 5)

Analyte	Spiked concentration [μg/l]	Mean recovery (rel.) * r* [%]	Range [%]
TEB‑OH	20	100	99–101
	150	100	99–100
TEB‑COOH	8	100	98–102
	60	101	99–103
PEN‑OH	5	100	91–107
	150	105	103–106
PEN‑COOH	5	106	100–106
	150	107	102–107

### 11.3 Matrix effects

In order to determine the effects of different urine matrices on the analytical results, six individual urine samples were each spiked with 20 μg or 150 μg of TEB‑OH/l, 8 μg or 60 μg of TEB‑COOH/l, 5 μg or 150 μg of PEN‑OH/l, and 5 μg or 150 μg of PEN‑COOH/l, then processed and analysed. The results are presented in Table 12.

**Tab.12:** Within-day precision data and relative recoveries for the determination of TEB‑OH, TEB‑COOH, PEN‑OH, and PEN‑COOH in individual urine samples (n = 6)

Analyte	Spiked concentration [μg/l]	Standard deviation (rel.) *s_w_* [%]	Mean recovery (rel.) *r* [%]	Range [%]
TEB‑OH	20	1.9	101	98–104
	150	1.1	99	97–101
TEB‑COOH	8	1.6	100	97–102
	60	1.2	100	98–101
PEN‑OH	5	1.3	101	99–103
	150	1.2	100	98–102
PEN‑COOH	5	1.4	100	97–102
	150	0.9	99	98–100

### 11.4 Limits of detection and quantitation

The detection limits for the determination of tebuconazole and penconazole metabolites were ascertained from the threefold signal-to-noise ratio. The quantitation limits (LOQs) of the analytes were calculated according to the equation:

LOQ = (5 × SE_*q*_ + *q*) ÷ *m*

where SE_*q*_ is the standard error of the intercept *q* and *m* is the slope of the regression line (according to Miller and Miller 2005).

The detection and quantitation limits calculated for the analytes are listed in Table 13.

**Tab.13:** Limits of detection and quantitation for the determination of TEB‑OH, TEB‑COOH, PEN‑OH, and PEN‑COOH in urine

Analyte	Detection limit [μg/l]	Quantitation limit [μg/l]
TEB‑OH	0.2	0.3
TEB‑COOH	0.1	0.3
PEN‑OH	0.5	1.0
PEN‑COOH	0.5	1.0

### 11.5 Sources of error

To extract the analytes from the matrix, this method uses online sample extraction with a TurboFlow^TM^ column. Even though a TurboFlow^TM^ column can be used in LC devices from any manufacturer, its full potential can only be realised with the Transcend TLX systems from Thermo Fisher Scientific. Such a system was used during method development.

In comparison to offline workup, which was also tested, the use of the TurboFlow system led to a significant reduction in blank values and, consequently, lower limits of detection.

After injecting samples with high analyte concentrations, analyte carryover was observed during method development. This carryover effect was counteracted by injecting ultra-pure water, e.g. after the most highly concentrated calibration standards. The verifiers of the method solved the carryover problem by injecting significantly smaller sample volumes into their UPLC system. Accordingly, only 5 μl instead of 50 μl were used in method verification, without compromising method sensitivity (see Appendix).

At the time of method development, no isotope-labelled, structurally identical compounds were commercially available as ISTDs for TEB‑OH, TEB‑COOH, PEN‑OH, and PEN‑COOH. For this reason, tebuconazole‑D_6_ was used as the ISTD for TEB‑OH and TEB‑COOH, and penconazole‑D_7_ as the ISTD for PEN‑OH and PEN‑COOH. These standards were only partially similar to the analytes in terms of their chemical structures (see Figure 1), but exhibited similar retention times to the analytes on the analytical column used here (see Table 8 as well as Figures 3 and 4).

External verification of the method was conducted using a UPLC system from Waters GmbH (Eschborn, Germany) in combination with a TurboFlow^TM^ column from Thermo Fisher Scientific GmbH (Dreieich, Germany). With this instrument, a matrix effect was ascertained – especially when quantifying the penconazole metabolites – which could not be sufficiently compensated by the use of penconazole‑D_7_ as ISTD and which negatively influenced method performance. For this reason, structurally identical, deuterated ISTDs for PEN‑OH and PEN‑COOH were synthesised (see Appendix for details) and used to standardise the signals for PEN‑OH and PEN‑COOH. Using PEN‑OH‑D_6_ and PEN‑COOH‑D_4_ as ISTDs, in addition to adjusting the chromatography (see Appendix), led to considerably improved recovery for the penconazole metabolites. However, the quantification of PEN‑OH was still interfered in some samples due to poorly separated matrix components. In this cases, the use of the qualifier trace made it possible to correctly quantify the analyte.

Field studies have shown an extremely high interindividual variability in the conjugate fractions for both penconazole and tebuconazole metabolites (Mercadante et al. 2014, 2016). Consequently, it is important when applying this method to ensure a well-adjusted and highly functional hydrolysis of the conjugates.

## 12 Discussion of the method

The aim of method development was a rapid and robust analytical method that enables the simultaneous determination of the most important metabolites of tebuconazole and penconazole in a single analytical run (Mercadante et al. 2014) The use of the TurboFlow technique for sample purification made it possible to reduce both work time and solvent consumption.

The use of tebuconazole‑D_6_ and penconazole‑D_6_ as ISTDs generally allowed for the compensation of analytical fluctuations. The robustness of the developed analytical method was confirmed by the analysis of spiked, individual urine samples: No matrix effect was observed when quantifying the analytes in various urine matrices.

The determination of tebuconazole and penconazole metabolites without TurboFlow technology used in method verification proved to be a viable alternative for laboratories without a TurboFlow device when using structurally identical deuterated ISTDs. Further examples of determination without TurboFlow technology can be found in the literature. In the analytical method developed by Sams et al. (2016) for the determination of penconazole and its metabolites, measurement by LC-MS/MS was also performed after enzymatic hydrolysis with *β‑*glucuronidase (*Helix pomatia*). A detection limit of 0.25 μg/l was reported for PEN‑COOH. In general, the methods described in the literature only ­detect either tebuconazole or penconazole metabolites, predominantly applying LC-MS/MS. These methods usually achieved comparable or slightly lower detection and quantitation limits (Alcalá et al. 2024; Bustamante et al. 2025; ­Rodríguez-Zamora et al. 2024; Sams et al. 2016). Šulc et al. (2022) published a detection limit for TEB‑OH that is six times lower. However, the method described here allows the two main metabolites of tebuconazole and penconazole to be detected simultaneously for the first time.

Instruments used

Method development: LC‑MS/MS system consisting of a TurboFlow system with two gradient pumps, an eluent degasser, and an autosampler (Thermo Fisher Scientific S.p.A., Rodano, Italy) and a tandem mass‑spectrometric detector (TSQ Quantum Access, Thermo Fisher Scientific S.p.A., Rodano, Italy)

Method verification: ACQUITY UPLC H-Class System (Waters GmbH, Eschborn, Germany) consisting of a quaternary pump (ACQ H-Class QSM Plus), a binary pump (UPLC Binary SOL MGR), an autosampler (ACQ H-Class FTN H-Plus) and a column manager (ACQUITY UPLC CM A) with triple-quadrupole mass spectrometer (Xevo TQ XS, Waters GmbH, Eschborn, Germany)

## Figures and Tables

**Fig.6:**
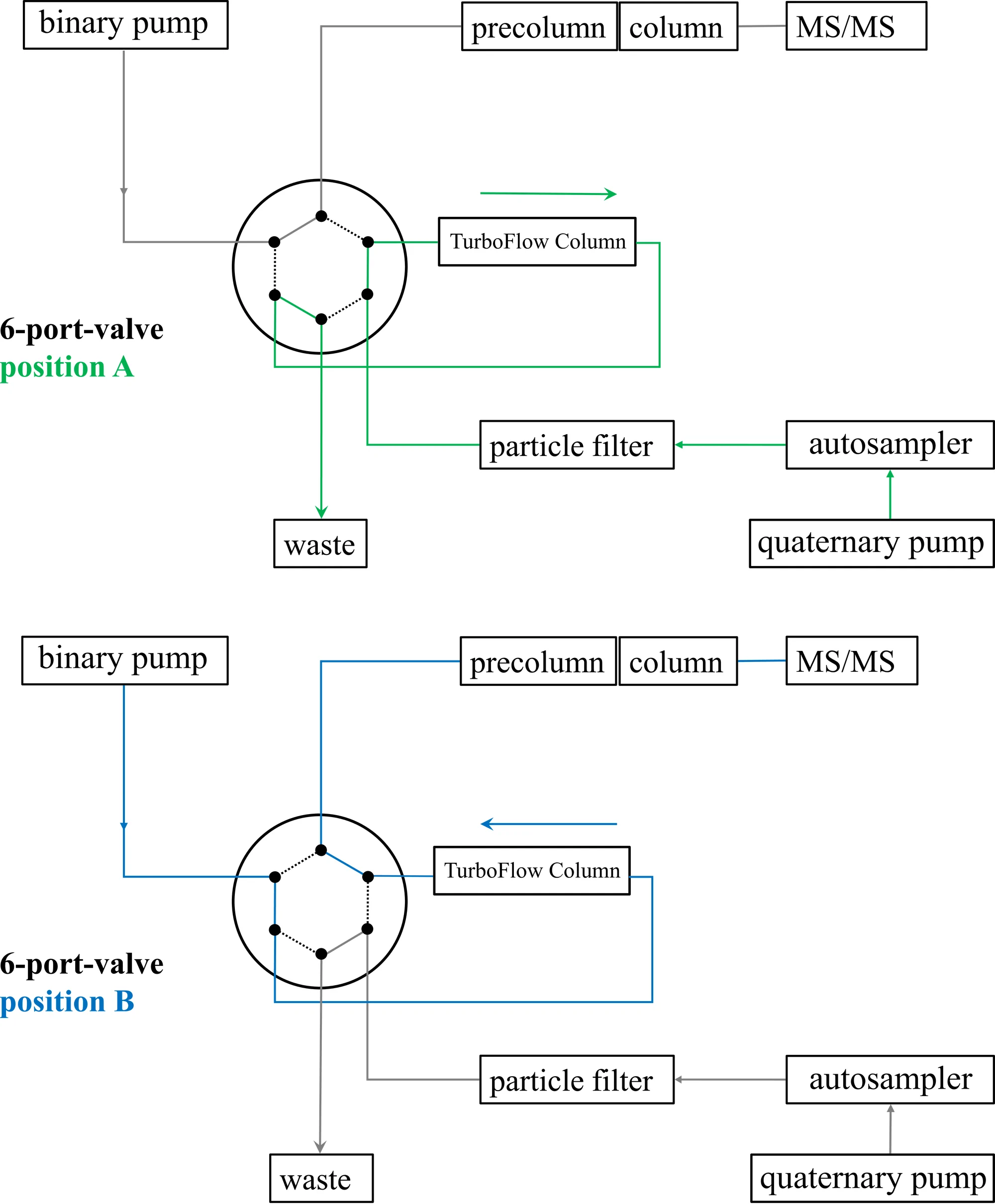
Scheme of the LC‑MS/MS system and illustration of the valve positions A (green) and B (blue) of the six-port valve (Kuhlmann et al. 2025)

**Fig.7:**
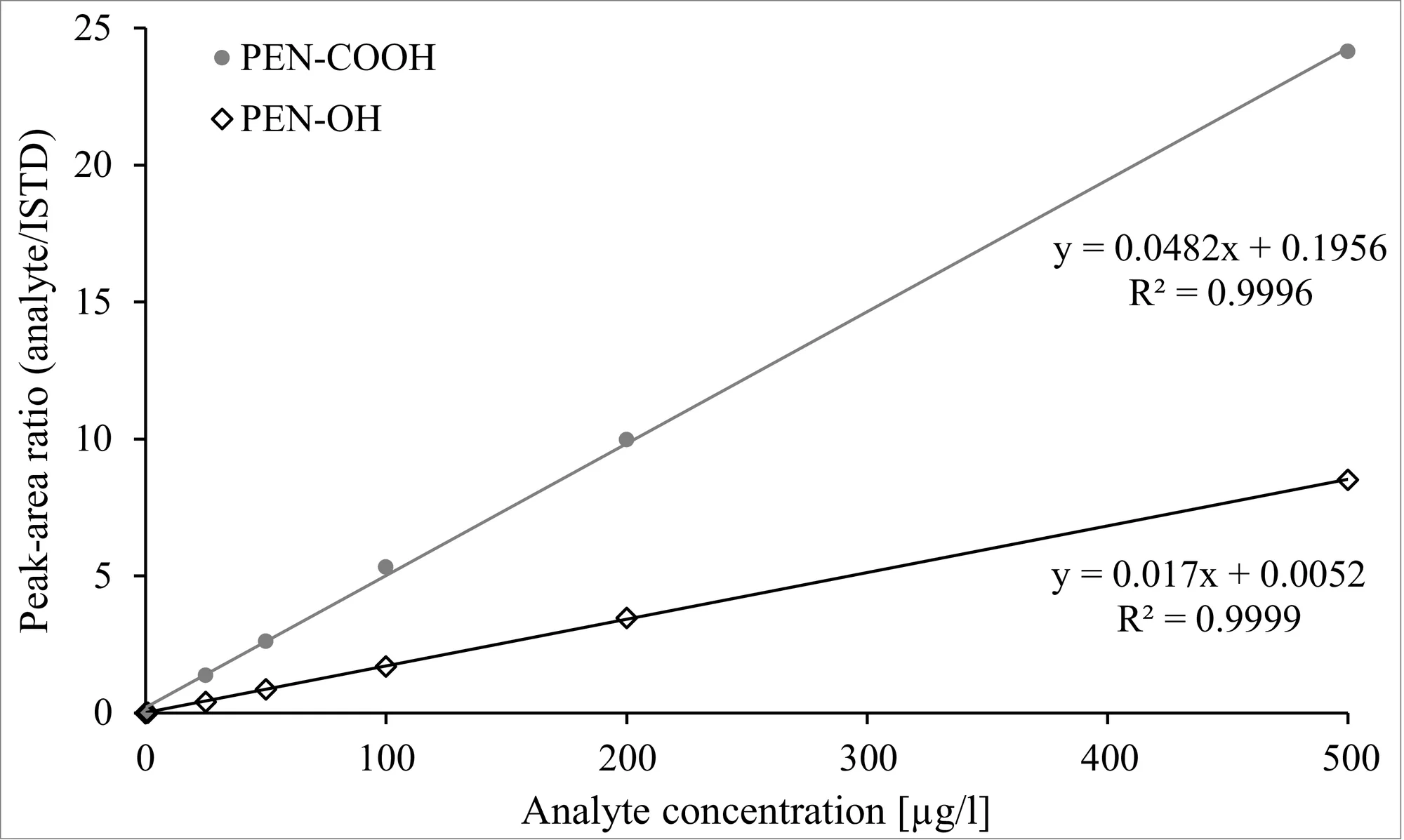
Calibration curves for the determination of PEN‑OH and PEN-COOH in urine using structurally identical, deuterated ISTDs

## Appendix

For external validation, a UPLC system from Waters GmbH (Eschborn, Germany) in combination with a TurboFlow^TM^ column from Thermo Fisher Scientific GmbH (Dreieich, Germany) was used. As a result, it was necessary to adjust the chromatographic parameters for enrichment, analyte transfer, and analyte separation. The associated operational parameters are listed below to facilitate the use of this method for users with no access to a Transcend TLX system.

### Equipment, chemicals, and solutions

#### Equipment

- LC system (ACQUITY UPLC H‑Class System) comprised of a quaternary pump (ACQ H‑Class QSM Plus), a binary pump (UPLC Binary SOL MGR), an autosampler (ACQ H‑Class FTN H‑Plus), and a column manager (ACQUITY UPLC CM A) (e.g. Waters GmbH, Eschborn, Germany)
- Triple-quadrupole mass spectrometer (e.g. Xevo TQ XS, Waters GmbH, Eschborn, Germany)
- In-line filter (e.g. No. 205000343, ACQUITY Column In-Line Filter Waters Critical Clean^TM^, Waters GmbH, Eschborn, Germany)
- Enrichment column (e.g. No. CH‑953288, TurboFlow^TM^‑Cyclone column, length: 50 mm, inner diameter: 0.5 mm, Thermo Fisher Scientific GmbH, Dreieich, Germany)
- Precolumn (e.g. No. 25403‑012101, Hypersil precolumn, 3 μm, 10 mm × 2.1 mm, Thermo Fisher Scientific GmbH, Dreieich, Germany)
- Analytical column (e.g. No. 25403‑052130, Hypersil Gold PFP, 3 μm, 50 × 2.1 mm, Thermo Fisher Scientific GmbH, Dreieich, Germany)

#### Chemicals

- *β‑*Glucuronidase/arylsulfatase from *Helix pomatia* (glucuronidase activity ≥ 100 000 U/ml) (e.g. No. BGALA‑RO, Roche Diagnostics Deutschland GmbH, Mannheim, Germany)
- 4-(2,4-Dichlorophenyl)-5-(1*H*-1,2,4-triazol-1-yl)pentan-1,1,2,2,3,3-D_6-1-ol (PEN‑OH‑D6)_ (e.g. custom synthesis, Max Planck Institute for Multidisciplinary Sciences, Facility for Synthetic Chemistry, Göttingen, Germany)
- 4-(2,4-Dichlorophenyl)-5-(1*H*-1,2,4-triazol-1-yl)pentanoic-2,2,3,3-D_4_ acid (PEN‑COOH‑D_4_) (e.g. custom synthesis, Max Planck Institute for Multidisciplinary Sciences, Facility for Synthetic Chemistry, Göttingen, Germany)

#### Solutions

- Eluent A (water : methanol : acetic acid (94.5 ∶ 5 ∶ 0.5; v/v/v))
  In a 500‑ml volumetric flask, 250 ml of ultra-pure water are placed. Subsequently, 25 ml of methanol and 2.5 ml of acetic acid are added and the flask is made up to the mark with ultra-pure water.

The solution is stable at room temperature for at least three months.

- Eluent C (acetonitrile : isopropanol (60 : 40; v/v))
  In a 500‑ml volumetric flask, 300 ml of acetonitrile are placed. Subsequently, the flask is made up to the mark with isopropanol.
- Eluent D (0.03% ammonium hydroxide in acetonitrile)
  In a 1-l volumetric flask, 500 ml acetonitrile are placed and 1 ml of the 30% ammonium hydroxide solution is added. The flask is then made up to the mark with acetonitrile.
- Eluent E (0.5% acetic acid in water)
  In a 500‑ml volumetric flask, 250 ml of ultra-pure water are placed and 2.5 ml of acetic acid are added. Subsequently, the flask is made up to the mark with ultra-pure water.

The solution is stable at room temperature for at least three months.

#### Internal standards (ISTDs)

- PEN‑OH‑D_6_ stock solution (1000 mg/l)
  About 5 mg of PEN‑OH‑D_6_ are weighed exactly into a 5‑ml volumetric flask and dissolved in acetonitrile. The flask is then made up to the mark with acetonitrile.
- PEN‑COOH‑D_4_ stock solution (1000 mg/l)
  About 5 mg of PEN‑COOH‑D_4_ are weighed exactly into a 5‑ml volumetric flask and dissolved in acetonitrile. The flask is then made up to the mark with acetonitrile.
- ISTD spiking solution (5 mg/l)
  10 μl of the PEN‑OH‑D_6_ stock solution, 10 μl of the PEN‑COOH‑D_4_ stock solution, and 980 μl of methanol are pipetted to a millilitre of the tebuconazole‑D_6_/penconazole‑D_7_ spiking solution (10 mg/l, see Section 4.4).

The PEN‑OH‑D_6_ and PEN‑COOH‑D_4_ stock solutions as well as the ISTD spiking solution are stored in the dark at −20 °C. Under these conditions, the solutions are stable for at least six months.

### Specimen collection and sample preparation

#### Sample preparation

Sample preparation corresponds with the procedures described in Section 5.2. To ensure more precise pipetting, 10 μl of the lower concentrated ISTD spiking solution (5 mg/l) are added to the samples.

### Operational parameters

The method was verified on an ACQUITY UPLC H‑Class System (Waters GmbH, Eschborn, Germany) comprised of a quaternary pump (ACQ H‑Class QSM Plus), a binary pump (UPLC Binary SOL MGR), an autosampler (ACQ H‑Class FTN H‑Plus), and a column manager (ACQUITY UPLC CM A) as well as a triple-quadrupole mass spectrometer (Xevo TQ XS, Waters GmbH, Eschborn, Germany).

The quaternary pump (loading pump) was used to load the sample onto the TurboFlow^TM^ column for enrichment. The binary pump (elution pump) serves the purpose of separating the sample extracts on a conventional analytical column (Figure 6).

#### Liquid chromatography

**Table:**

Enrichment column:	TFLC Cyclone, 50 × 0.5 mm
Precolumn:	Hypersil precolumn, 3 μm, 10 mm × 2.1 mm
Analytical column:	Hypersil Gold PFP, 3 μm, 50 × 2.1 mm
Mobile phases of quaternary pump P1:	Eluent A: 0.5% acetic acid in water Eluent B: methanol Eluent C: acetonitrile : isopropanol (60 : 40; v/v) Eluent D: 0.03% ammonium hydroxide in acetonitrile
Mobile phases of binary pump P2:	Eluent E: 0.5% acetic acid in water Eluent F: methanol
Flow rate of quaternary pump P1:	see Table 14
Flow rate of binary pump P2:	see Table 15
Column temperature:	Room temperature
Injection volume:	5 μl
Gradient programme:	see Tables 14 and 15
Valve-switching programme:	see Table 16

**Tab.14:** Gradient programme of the quaternary pump P1 (external verification)

Time [min]	Flow rate [ml/min]	Eluent A [%]	Eluent B [%]	Eluent C [%]	Eluent D [%]
Start	1.00	95	5	–	–
1.50	1.00	95	5	–	–
1.58	0.15	95	5	–	–
3.30	0.15	–	–	100	–
3.75	1.00	–	–	100	–
4.92	1.00	–	–	100	–
5.00	1.00	–	–	–	100
6.00	1.00	–	–	–	100
6.10	1.00	30	70	–	–
7.00	1.00	30	70	–	–
7.50	1.00	95	5	–	–
10.0	1.00	95	5	–	–

**Tab.15:** Gradient programme of the binary pump P2 (external verification)

Time [min]	Flow rate [ml/min]	Eluent E [%]	Eluent F [%]
Start	0.50	40	60
1.00	0.50	40	60
4.00	0.50	40	60
4.10	0.50	20	80
5.00	0.50	0	100
8.00	0.50	0	100
8.50	0.50	40	60
10.0	0.50	40	60

**Tab.16:** Valve-switching programme (external verification)

Time [min]	Valve position	Description
Initial	A	Preconcentration and purification of analytes on enrichment column
1.50–3.75	B	Transfer of analytes from enrichment column to analytical column in backflush mode
3.75–10.0	A	Chromatographic separation of analytes, rinsing and equilibration of enrichment column

It is recommended to add a particle filter in front of the enrichment column in order to protect the columns and instrument components. All other parameters must be optimised according to manufacturer specifications.

#### Tandem mass spectrometry

**Table:**

Ionisation mode:	Positive electrospray ionisation (ESI+)
Capillary voltage:	3.5 kV
Source temperature:	150 °C
Desolvation temperature:	600 °C
Cone-gas flow:	150 l/h
Desolvation-gas flow:	1000 l/h
Nebuliser gas:	Nitrogen, 7.00 bar
Collision gas:	Argon, 0.15 ml/min

### Analytical determination

Of the processed sample, 5 μl are injected into the LC‑MS/MS system. The analytes are identified by their characteristic retention times and specific mass transitions. The retention times of the analytes and ISTDs as well as the ion traces used are presented in Table 17.

**Tab.17:** Retention times, ion transitions, and collision energy for the determination of TEB‑OH, TEB‑COOH, PEN‑OH und PEN‑COOH in urine

Analyte or ISTD	Retention time [min]	Ion trace (*m/z*)		Cone voltage [V]	Collision energy [eV]
		Q1	Q3		
TEB‑OH	2.74	324	70^a)^	20	18
		324	179	20	22
		324	125	20	40
TEB‑COOH	2.68	338	125^a)^	20	42
		338	70	20	18
		338	163	20	22
Tebuconazole‑D_6_	3.42	314	72^a)^	20	20
		314	125	20	36
		314	154	20	26
PEN‑OH	2.25	300	70^a)^	20	14
		300	143	20	32
		300	159	20	28
PEN‑COOH	2.24	314	159^a)^	18	36
		314	185	18	24
		314	70	18	16
PEN-OH‑D_6_	2.25	306	70^a)^	38	14
		306	148	38	36
		306	160	38	34
PEN-COOH‑D_4_	2.24	320	70^a)^	18	20
		320	151	18	36
		320	159	18	38

a) quantifier

### Calibration and calculation of the analytical results

For the tebuconazole metabolites, calibration and calculation of the analytical results is carried out as described in Sections 8 and 9. For the penconazole metabolites, the calibration curves are created by plotting the peak-area ratios of PEN‑OH or PEN‑COOH and the corresponding ISTDs (PEN‑OH‑D_6_ or PEN‑COOH‑D_4_) against the respective concentration of the calibration standard. The linear working ranges for the determination of tebuconazole and penconazole metabolites corresponded to the concentration ranges specified by the method developers. Exemplary calibration curves for the determination of PEN‑OH and PEN‑COOH using structurally identical, deuterated ISTDs are shown in Figure 7.

### Quality control

Quality assurance of the analytical results was carried out as stipulated in the guidelines of the *Bundesärztekammer* (German Medical Association) and in a general chapter published by the Commission (Bader et al. 2010; Bundesärztekammer 2023).

### Evaluation of the method

The verifiers of the method comprehensively validated the entire method using their own UPLC‑MS/MS system. Both the precision data and the analyte recoveries corresponded to the good reliability data reported by the method developers. The analysis of spiked individual urines during external verification also yielded precise and accurate results with no indication of a matrix effect.

During external method verification, the detection and quantitation limits were ascertained using the calibration-curve method. The equidistant ten‑point calibration curves spanned a concentration range from 0.1–1.0 μg/l. The limits of detection and quantitation for the tebuconazole metabolites given in Table 18 were similar to the values reported by the method developers, whereas better values were achieved for the detection and quantitation limits of the penconazole metabolites.

**Tab.18:** Limits of detection and quantitation for the determination of TEB‑OH, TEB‑COOH, PEN‑OH, and PEN‑COOH in urine

Analyte	Detection limit [μg/l]	Quantitation limit [μg/l]
TEB‑OH	0.07	0.21
TEB‑COOH	0.08	0.26
PEN‑OH	0.05	0.16
PEN‑COOH	0.09	0.27

Overall, it can be concluded that the adapted UPLC‑MS/MS method also enables a precise and accurate quantification of the most important tebuconazole and penconazole metabolites.
